# Optimization of an Ultrasound-Assisted Extraction for Simultaneous Determination of Antioxidants in Sesame with Response Surface Methodology

**DOI:** 10.3390/antiox8080321

**Published:** 2019-08-19

**Authors:** Dandan Wang, Liangxiao Zhang, Yueqing Xu, Xin Qi, Xuefang Wang, Xiupin Wang, Qi Zhang, Peiwu Li

**Affiliations:** 1 Oil Crops Research Institute, Chinese Academy of Agricultural Sciences, Wuhan 430062 China; 2 Key Laboratory of Biology and Genetic Improvement of Oil Crops, Ministry of Agriculture and Rural Affairs, Wuhan 430062, China; 3 Laboratory of Quality and Safety Risk Assessment for Oilseed Products (Wuhan), Ministry of Agriculture and Rural Affairs, Wuhan 430062, China; 4 Quality Inspection and Test Center for Oilseed Products, Ministry of Agriculture and Rural Affairs, Wuhan 430062, China; 5 Key Laboratory of Detection for Mycotoxins, Ministry of Agriculture and Rural Affairs, Wuhan 430062, China

**Keywords:** antioxidants, ultrasound-assisted extraction, liquid chromatography-tandem mass spectrometry (LC-MS/MS), sesame, simultaneous detection, response surface methodology

## Abstract

Sesame is a nutritional agricultural product with medicinal properties. Accurate determination of micronutrients is important for the improvement of sesame quality and nutrition assessments. Our previous study showed that 10 antioxidants—d-homoproline, vitamin B_2_, coniferyl aldehyde, hesperidin, phloretin, N-acetyl-l-leucine, l-hyoscyamine, ferulic acid, 5-methoxypsoralen, and 8-methoxypsoralen—in sesame were potential characteristic nutrients in sesame. Herein, simultaneous detection of 10 different types of antioxidants was developed by using ultrasound-assisted extraction coupled with liquid chromatography-tandem mass spectrometry (UAE-LC-MS/MS) with the help of response surface methodology. The significant variables and levels were screened and optimized by combining the single factor experiment, Plackett–Burman test, and Box–Behnken design. The optimal conditions for extraction of target antioxidants in sesame were methanol solution of 75.0%, liquid-to-material ratio of 20:1 (mL/g), extraction temperature of 50 °C, extraction power of 410.0 W, extraction time of 65 min. The total yield of targets was 21.74 μg/g under the optimized conditions. The mobile phase used was 0.1% formic acid in acetonitrile and 0.1% formic acid in water, and the column was a Thermo Syncronis C18 reverse phase column (100 mm × 2.1 mm, 3 μm). All targets required only one injection and could be quickly separated and assayed within 7 min. The limits of detection and limits of quantification for these 10 nutritional compounds ranged from 0.01 to 0.11 µg/kg and from 0.04 to 0.34 µg/kg, respectively. The validation results indicated that the method had reasonable linearity (*R*^2^ ≥ 0.9990), good recoveries (71.1%–118.3%), satisfactory intra-day precision (≤9.6%) and inter-day precision (≤12.9%), and negligible matrix effects (≤13.8%). This simultaneous quantification method was accurate, fast, and robust for the assessment of sesame nutrition.

## 1. Introduction

Sesame (*Sesamum indicum* L.) possesses high nutritional value and various physiological functions including antioxidant properties [1], detoxification, blood lipid regulation [2], liver protection, and anti-cancer activities [3]. Sesame has been used for health purposes since ancient times because it contains lipids and proteins, as well as sterols, polyphenols, vitamins, minerals, and lignans. Black sesame is more expensive than white sesame, but the differences of chemical composition between them remain unclear. Several components with significant differences between black and white sesame seeds, mainly including hesperidin, d-homoproline, vitamin B_2_, coniferyl aldehyde, 5-methoxypsoralen, phloretin, and l-hyoscyamine, were discovered in our previous study [4]. Therefore, it is necessary to accurately quantify these compounds with beneficial functions for further evaluation and nutritional sciences. 

Previous studies have shown that these specific compounds have great physiological activities and pharmacological values. Hesperidin is a flavonoid that can capture free radicals, chelate metal ions [5], enhance capillary toughness, reduce cholesterol [6], prevent cardiovascular diseases, and treat high blood pressure and myocardial infarction [7]. Hesperidin plays a strong part in anti-photoaging and skin protection by inhibiting the expression of matrix metalloproteinase-9 [8]. Vitamin B_2_ (riboflavin) is an indispensable intermediate in proteins, lipids, and glucose metabolic pathways [9]. The lack of vitamin B_2_ could cause a significant increase in the risk of skin and mucosal disorders and anemia [10]. It was recently shown that the appropriate amount of vitamin B_2_ intake could effectively prevent diabetes in elderly people [11]. N-acetyl-l-leucine, a powerful and common anti-vertigo drug, has an important influence on the repair of motor balance and the activity of the vestibulocerebellum [12]. It is also involved in the apoptosis process of cancer cells and the proliferation and migration of prostate cancer cells [13]. N-acetyl-l-leucine was enzymatically esterified with 2-acylglycerols to produce healthy new modified lipids to replace oils such as margarines and shortenings in foods [14]. Coniferyl aldehyde is a phenolic compound that can effectively scavenge free radicals, resist mutagenesis, fight cancer, and treat neurodegenerative diseases [15]. Coniferyl aldehyde can also inhibit the death of endothelial cells to reduce radiation-induced bowel disease and prevent cancers [16]. Phloretin is a chalcone with strong antioxidant properties in peroxynitrite scavenging, as well as cardioprotective and immune-enhancing effects [17]. Moreover, it inhibits lipid peroxidation to protect the skin [18]. Phloretin can reduce the endothelial dysfunction caused by hyperuricemia by co-inhibiting inflammation and uric acid uptake [19]. l-hyoscyamine is an alkaloid with anti-cancer and anti-oxidative properties. Lynch et al. demonstrated that l-hyoscyamine could be used as a reasonable cost-effective intravenous alternative to glucagon for duodenal antimotility during endoscopic retrograde cholangiopancreatography (ERCP) [20]. d-homoproline (pipecolinic acid) is a widely occurring amino-acid that can affect the receptor function of neurotransmitter-gated ion channels [21]. The 5-methoxypsoralen and 8-methoxypsoralen are both furocoumarins with an irreplaceable role in anti-microbial, anti-viral, and anti-tumor activity [22]. Ferulic acid is a polyphenol with good antioxidant and anti-aging functions, which can also lower cholesterol to prevent coronary heart disease and protect nerves [23]. In summary, 10 nutritional functional target components were identified for quantitative research by combining Chinese traditional pharmacy knowledge and current nutrition research.

Common methods for extracting biologically active ingredients include liquid–liquid extraction, solid phase extraction and supercritical extraction techniques. However, liquid–liquid extraction consumes large amounts of time and solvent with low extraction efficiency, while solid phase extraction greatly increases the cost of pretreatment and has strong specificity [24]. Novel extraction techniques, such as supercritical fluid extraction and deep eutectic solvent extraction, require high operational requirements and have poor practicality. Ultrasonic-assisted extraction technique is cheap, efficient, adjustable, applicable, and easy to operate versus other extraction methods [25]. The use of the ultrasonic-assisted extraction method not only increased the yield of the targets but also greatly improved the purity. Huang et al. showed that ultrasonic-assisted extraction method had higher extraction efficiency for flavonoids in *Eucommia ulmoides* leaves than heating extraction, microwave-assisted extraction, and enzymatic extraction [26]. These 10 compounds belong to different classes and have different physical and chemical properties. Therefore, a highly compatible extraction method is needed. Due to the diverse structures of the 10 metabolites and the large differences in physicochemical properties, it is difficult to obtain optimal extraction conditions in single factor experiments. Therefore, ultrasonic-assisted extraction technique is combined with Plackett–Burman design, Box–Behnken design, and response surface method to optimize the extraction conditions of these 10 nutrients and establish a fast, robust and sensitive UAE-HPLC-MS/MS method. The aim of this study was to develop an optimal ultrasonic-assisted extraction technology by adopting Plackett–Burman design and Box–Behnken design with response surface methodology. In addition, a rapid, robust, and sensitive HPLC-MS/MS method for the quantification of these 10 nutritional compounds in sesame seeds was established. Ten differential nutrients in sesame seeds were successfully extracted and analyzed under these conditions. It is of great significance for the evaluation, screening and quality improvement of sesame seeds.

## 2. Experimental

### 2.1. Reagents and Standards

Methanol (99.9% purity) was purchased from Thermo Fisher Scientific Co., Ltd. (Shanghai, China). Formic acid (95% purity) was purchased from Sigma-Aldrich Co., Ltd. (Shanghai, China). Acetonitrile (99.8% purity) was purchased from Beijing Bellingway Technology Co., Ltd. (Beijing, China). Ultra-pure water was obtained from a Milli-Q water purification system (Millipore Co., Ltd., Milford, MA, USA) and used to prepare all aqueous solutions.

Vitamin B_2_ (95% purity), N-acetyl-l-leucine (98% purity), phloretin (98% purity), d-homoproline (99% purity), 8-methoxypsoralen (98% purity), ferulic acid (99% purity) were all purchased from Shanghai Aladdin Biochemical Technology Co., Ltd. (Shanghai, China). Coniferyl aldehyde (98% purity) was gained from Sigma-Aldrich Co., Ltd. (Shanghai, China). 5-methoxypsoralen (98% purity) was obtained from Beijing Bellingway Technology Co., Ltd. (Beijing, China). l-hyoscyamine, hesperidin with a purity of >98% were purchased from Shanghai Yuanye Biotechnology Co., Ltd. (Shanghai, China). 

### 2.2. Preparation of Standard Solutions

Stock solutions were freshly prepared for all standards except vitamin B_2_ by accurately weighing out 5 ± 0.1 mg of standard substances and separately dissolving it in 10.0 mL methanol. Vitamin B_2_ was dissolved in ultra-pure water to prepare the stock solution. All standard stock solutions obtained the concentration of 500 μg/mL and were kept in dark brown calibrated flasks at −20 °C and set aside. The working standard solutions were prepared by diluting the standard stock solution with the corresponding solvent. Single working standard solution was injected alone in order to identify their retention time, collision energy and ion fragmentation information. Mixed standard stock solutions with different concentrations were obtained by precisely adding appropriate amounts of individual standard stock solution in methanol. A seven-point calibration curve of each standard was subsequently built from mixed working standard solutions with different concentrations.

### 2.3. Samples

The 54 representative sesame seeds samples were purchased from different producing areas mainly including Shandong, Henan, Jiangxi, Hubei, Jilin, Liaoning, Jiangsu, Yunnan, Sichuan and other cities. The sesame seeds samples were ground for 60 s at a frequency of 40 Hz using 80350-CN grinder (Hamilton Beach Electric Co., Ltd., Shenzhen, China) and stored at −20 °C for later use. 

### 2.4. Extraction Process

The target extraction used an ultrasonic bath. The bath (DTC-27J, Dingtai Biochemical Technology Equipment Manufacturing Co., Ltd., Wuhan, China) was a rectangular container (volume 27 L) with adjustable temperature, power, time, and a fixed frequency of 40 kHz. Sesame seeds (0.20 g) were weighed into a 10-mL plastic centrifuge tube (Trading Company, Shanghai, China) with an AUW-220D electronic analytical balance (Shimadzu Company, Kyoto, Japan), and then stored in a 4 °C refrigerator for later use. Different concentrations of methanol solution were added to the weighed samples with a pipette (Eppendorf Company, Hamburg, Germany) according to the ratio of material-to-liquid required for the experiments. The solution was vortexed for 10 s on an XH-C vortex mixer (Future Instrument Manufacturing Co., Ltd., Changzhou, China) and then extracted in an ultrasonic bath. Samples were sonicated for different times with the required temperature controlled by a water bath. The extracts were then centrifuged at 5000 rpm for 10 min in a HITACHI CT6E centrifuge (HITACHI, Tokyo, Japan). 1 mL supernatant solution was filtered through a 0.22 μm organic phase filter (Millipore Co., Ltd., Milford, MA, USA) and collected in a 1.5-mL vial (Agilent Technology Co., Ltd., Palo Alto, CA, USA) for HPLC-MS/MS analysis.

### 2.5. Optimization of the Extraction Process Parameters

Black sesame seeds of the same variety were used to optimize the extraction conditions. The ultrasonic extraction process is affected by many factors, and the parameters were efficiently optimized by combining Plackett–Burman design and Box–Behnken design. The main extraction parameters that affect the extraction efficiency include liquid-solid ratio (X_1_), ultrasonic power (X_2_), ultrasonic time (X_3_), ultrasonic temperature (X_4_), methanol volume fraction (X_5_), and extraction times (X_6_). Each experiment was performed with 0.20 g sesame powders, and the effects of each factor were evaluated by analyzing the total content of all compounds. The yield was calculated as follows:

Y = 10^−3^*C * V/M (1)

where C is the concentration of each compound in sesame samples (ng/mL) as determined by HPLC-MS/MS; V is the extract volume measured (mL); M is the sample mass before extraction (g); 10^−3^ is a conversion multiple; and Y is the yield of each compound (μg/g).

#### 2.5.1. Single Factor Experiment

The total yield was used to represent the extraction efficiency considering the difference in the physical and chemical properties of each compound. The single factor experiment was used to determine the range of optimal parameters. A suitable liquid-solid ratio was an important parameter to improve the extraction yield and reduce the waste of solvent. A series of ratios were investigated in this study in order to evaluate the effect of the ratio of liquid-solid on the total extraction yield. Different concentrations (dilutions) of methanol were used for extraction. Ultrasonic power, ultrasonic time, ultrasonic temperature, and extraction times were also investigated. The common conditions selected were liquid-solid ratio of 10 mL/g, ultrasonic power of 300 W, ultrasonic time of 30 min, methanol solution of 70% as the extraction solvent, ultrasonic temperature of 45 °C, and one extraction.

#### 2.5.2. Plackett–Burman Design

Plackett-Burman (PB) design is widely used to estimate the main effects of factors and the screening of significant parameters [27]. Based on the single factor experiment, Plackett–Burman design was used to screen the important factors affecting the total extraction yield of the target compounds. We selected a design scheme for 6 factors (X_1–6_) with 12 experimental rounds to investigate the extraction factors. Each factor has high and low levels. Supplementary materials table S1 detailed the factors and levels of Plackett–Burman design. Table S2 displayed the design matrix and the experimental results. A first-order polynomial model was applied for PB experimental design as follows:

(2) $$Y=\beta o+\sum_{1}^{6}\beta_{i}X_{i}$$

where Y is the predicted value (total yield μg/g), βo is the intercept, β_i_ is the linear regression parameter estimate, and X_i_ is the coded level of the variable. This model is mainly applied to the selection and evaluation of significant (*p* < 0.05) important factors that affect the response. All of the experiments were done in triplicate.

#### 2.5.3. Box–Behnken Design

Box–Behnken (BB) design was used to further optimize the value and interaction effects of the factors that impact total extraction yield [28]. The data were subjected to quadratic polynomial regression fitting to establish a mathematical model of the independent variables. Meanwhile, the optimal extraction conditions were predicted by combining analysis of variance, the level of each factor, and the interaction evaluation results. A response surface analysis graph was obtained according to the regression equation [29]. In Box–Behnken design, high, medium, and low variable values are coded (−1, 0, and 1), and a total of 15 sets of experiments using a three-factor and three-level design matrix are adopted. Table S3 showed the independent variables and levels, and Table S4 presented the specific experimental design. The general form of the fitted quadratic polynomial equation is as follows:

Y = A_0_ + B_1_X_1_ + B_2_X_2_ + B_5_X_5_ + C_12_X_1_X_2_ + C_15_X_1_X_5_ + C_25_X_2_X_5_ + D_1_X_1_^2^ + D_2_X_2_^2^ + D_5_X_5_^2^ (3)

where Y refers to the predicted value (total yield μg/g); A_0_ refers to the intercept of the equation; B_1_, B_2_, and B_5_ are the linear coefficients of the liquid-solid ratio (X_1_), ultrasonic power (X_2_), and methanol concentration (X_5_), respectively; C_12_, C_15_, and C_25_ are the interaction coefficients among the variables; and D_1_, D_2_, and D_5_ are the square coefficients of these three variables.

### 2.6. HPLC-MS/MS Conditions

The HPLC-MS/MS apparatus consisted of an Accela HPLC system coupled to a triple quadrupole mass spectrometer Thermo TSQ Quantum Ultra EMR (Thermo Fisher Scientific, MA, USA). The 10 nutritional compounds were separated by HPLC using a solvent system consisting of 0.1% formic acid in acetonitrile (solvent A; *V*/*V*) and 0.1% formic acid in water (solvent B; *V*/*V*) along with a Thermo Syncronis C18 column (100 mm × 2.1 mm i.d., 3 µm). The gradient elution program for HPLC-MS/MS analysis was as follows: 0 min, 5% A; 0.5 min, 5% A; 6 min, 95% A; 8 min, 95% A; 8.01 min, 5% A; and 10.5 min, 5% A. The injection volume was 10 μL, and the flow rate was 200 µL/min. The column temperature was held near 35 °C to improve the reproducibility of the retention time.

The MS/MS acquisition used a Thermo TSQ Quantum MS triple quadrupole mass spectrometer equipped with an electrospray ionization interface operating in positive ion mode (ESI^+^). The mass spectrometric detection conditions were as follows: spray voltage, 4.0 kV; capillary temperature, 320 °C; vaporizer temperature, 300 °C; sheath gas pressure (nitrogen, purity 99.9%), 40 arb; auxiliary gas pressure (nitrogen, purity 99.9%), 15 arb; collision gas (argon, purity 99.999%), 1.5 mTorr; and cycle time, 0.6 s. For each target analyte, two characteristic ions were selected for precise quantification while optimizing the corresponding collision energy and tube lens. The standards were injected directly into the mass spectrometer to find extremely precise ion fragments, collision energy and tube lens, which resulted in high sensitivity and low limit of quantitation. The detailed mass spectrometric parameters include the ion polarity, optimum collision energy, as well as parent ions and product ions (Table 1).

### 2.7. Peak Identification

In selected reaction monitoring mode, the peaks of the different analytes were specifically identified by observing the retention time (RT) and monitoring precursor ions and product ions. The maximum peak area can be improved by optimizing the ion-spray and relevant MS parameters. Method establishment, data acquisition, and data processing were all conducted with Xcalibur software version 2.0.7 (Thermo Fisher Scientific, Waltham, MA, USA).

### 2.8. Statistical Analysis

Instrumental quality and method feasibility parameters, such as the precision, linearity, stability, sample recovery, calibration curve, reproducibility, limit of detection, and limit of quantitation, were analyzed to evaluate the performance of the method. All of the sesame seed samples were measured in triplicate, and the results were presented as average values ± standard deviations (SD). The measurements were completed with an external standard method. Data analysis was carried out by using Xcalibur software version 2.0.7 (Thermo Fisher Scientific, Waltham, MA, USA). The PB and BB experimental design and the data analysis were achieved with SAS 9.2.

## 3. Results and Discussion

### 3.1. Optimization of Extraction Conditions by Single Factor Experiments

To improve the efficiency of ultrasound-assisted extraction of these nutritional compounds from sesame seeds, the parameters, including the liquid-solid ratio, ultrasonic temperature, ultrasonic time, ultrasonic power, methanol volume fraction, and extraction times, were optimized. The extraction efficiency of these 10 nutritional compounds was primarily investigated by comparing the total yield of all of the compounds.

#### 3.1.1. Effect of Methanol Volume Fraction

Methanol was used as the extraction solvent because most of the analytes have been extracted with methanol [30]. The effect of extraction solvent on extraction efficiency was evaluated with 10%, 30%, 50%, 70%, and 90% methanol. The total yield of the 10 nutritional compounds changed with methanol concentration (supplementary materials Figure S1a); 70% methanol gave the best yield and was used for all of the subsequent experiments.

#### 3.1.2. Effect of Extraction Time

The effect of extraction time on extraction efficiency was researched by increasing the time from 5 to 85 min at 45 °C. The total yield increased rapidly in the first 50 min and then slowly increased and finally even decreased (Figure S1b). This could be because of oxidation or decomposition of some compounds as the extraction time increased. We concluded that the highest efficiency was achieved at 65 min. The optimal ultrasonic extraction time was determined to be 65 min.

#### 3.1.3. Effect of Liquid-Solid Ratio

Different liquid-solid ratios (5/1, 10/1, 15/1, 20/1, 25/1, and 30/1 mL/g) were examined. The effect of the liquid-material ratio on the extraction efficiency of the compounds was shown in Figure S1c. The total extracts gradually increased as more extractant methanol was added but plateaued when the liquid-to-material ratio reached 20:1 (mL/g). A ratio of 20:1 (mL/g) gave the highest total yield and was used for all subsequent experiments.

#### 3.1.4. Effect of Ultrasonic Power

Ultrasonic power was studied at 100 W, 250 W, 400 W, 550 W, and 700 W. The increase in ultrasonic power could significantly improve the total yield of these 10 nutrient components through 400 W (Figure S1d). However, the total yield decreased over 400 W. The appropriate power of ultrasonic treatments might promote cell rupture and facilitate the release of the target compound. Excessive ultrasonic power might also destroy the structure of the compounds and even increase the dissolution of fat-soluble impurities. This could also affect extraction efficiency. Therefore, 400 W of ultrasonic power was used for the extraction of these 10 analytes.

#### 3.1.5. Effect of Ultrasonic Temperature

Ultrasonic temperature can affect the total yield of these 10 nutrients by changing the solubility and stability of the different compounds. The influence of ultrasonic temperature on total yield was examined at 20 °C, 35 °C, 50 °C, 65 °C, and 85 °C via a water bath. Figure S1e showed that the extraction was stimulative when the extraction temperature was below 50 °C, and the total yield decreased gradually after this point. Thus, 50 °C was used for all subsequent experiments.

#### 3.1.6. Effect of Extraction Times

The effect of extraction times on extraction efficiency was shown in Figure S1f. A total of four extractions was performed to investigate whether the extraction was sufficient. The results indicated that there was no significant difference in the total yield from the different extraction times. One extraction was selected in this experiment to save time, reduce waste, and improve efficiency.

### 3.2. Selecting Important Parameters via Plackett–Burman Design

The Plackett–Burman design is an effective two-level test design that can identify factors that have a significant impact on the results with fewer trials, improving test efficiency and avoiding wasted test resources [27,31]. Based on the significant factors of Plackett–Burman screening, Box–Behnken response surface design quickly fits the nonlinear model to obtain the best process [28]. Plackett–Burman design can only evaluate the main effect of each factor, while Box–Behnken design can further examine the interaction effect between factors. The experimental design advantage is to examine the main effects and interaction effects between factors quickly and accurately with the least resource consumption.

Regression analysis and analysis of variance were carried out using SAS 9.2 according to the design and experimental results of Plackett–Burman test in Table S2. The Plackett–Burman test results were shown in Table S5. A first-order regression equation for the impact of the various factors on the total yield of 10 target compounds was obtained based on regression analysis: Y = 12.32 + 1.32X_1_ − 1.00X_2_ − 0.16X_3_ + 0.45X_4_ − 2.56X_5_ + 0.22X_6_. A lower probability (*p* > F) suggests a more significant variable correlation. This also implies a more consistent regression model and test results. The *p*-value of the entire model was 0.0032 (Pr > F < 0.01), indicating that the regression equation was very significant. When the determination coefficient value (*R*^2^) is close to 1, it means that the actual value and the predicted value are related and the model fitting is effective [32]. The *R*^2^ value of the model was 0.9547, and the adjustment determination coefficient (*R*^2^_Adj_) was 0.9004. This indicated that the equation could explain a change of 90.04% in response value, and the equation fitted well. The effects of X_1_ (liquid-solid ratio), X_2_ (ultrasonic power), and X_5_ (methanol concentration) on the total extraction rate of 10 target compounds were significant (*p* < 0.05); the other factors were not significant. Therefore, the variables X_1_ (liquid-solid ratio), X_2_ (ultrasonic power), and X_5_ (methanol concentration) were selected for further study.

### 3.3. Optimizing Important Parameters by Box-Behnken Design and Response Surface Methodology

Regression fitting analysis and analysis of variance were performed according to the design and experimental data of Box–Behnken test (Table S4). The detailed analysis results of Box–Behnken test are shown in Table S6. The quadratic multiple regression equations of three significantly important independent variables were obtained: Y = −151.97 + 4.51X_1_ + 0.30X_2_ + 172.53X_5_ + 9.36*10^−4^X_1_X_2_ − 0.16X_1_X_5_ + 0.01X_5_X_2_ − 0.11X_1_^2^ − 3.95*10^−4^X_2_^2^ − 115.92X_5_^2^.

The “Prob > F” value of the total model was 0.0002 (<0.01), indicating that the quadratic equation model is extremely significant. The “Pr>|t|” values of the three factors of liquid-solid ratio (Pr>|t| = 0.0008), ultrasonic power (Pr>|t| = 0.0002), and methanol volume fraction (Pr>|t| = 0.0117) were all less than 0.05, which indicated that their impacts on analyte extraction were significant. At the corresponding confidence level, the F-value of the fitted model exceeds by 1.5 times that of the F-value listed in the ANOVA table, indicating that the model is significant [33]. The high F value and *R*^2^ value of the total model (F = 51.48 > 1.5 F_9,5_, *R*^2^ = 0.9893) also indicated that the multivariate regression relationship between the dependent variable and the three independent variables was significant and the model fitted the actual experimental results nicely.

The linear and quadratic of the regression model was extremely significant (Pr > F < 0.01). Lack of fit is an important datum to evaluate the reliability of regression equations. If the *p*-value for the lack of fit is significant (<0.05), then the equation is not well-fitted and needs to be adjusted. Conversely, if it is not significant (>0.05), the equation is well fitted and can be used for promotion and application. The lack of fit of the quadratic regression model was insignificant *(p* = 0.0936 > 0.05), demonstrating the equation fitted reasonably well. In addition, the coefficient of variation indicating the accuracy and repeatability of the model (CV = 2.9571) was acceptable. Therefore, it is suitable to adopt the model to analyze and predict the total yield of these 10 target compounds under different ultrasonic extraction conditions.

**Figure 1:**
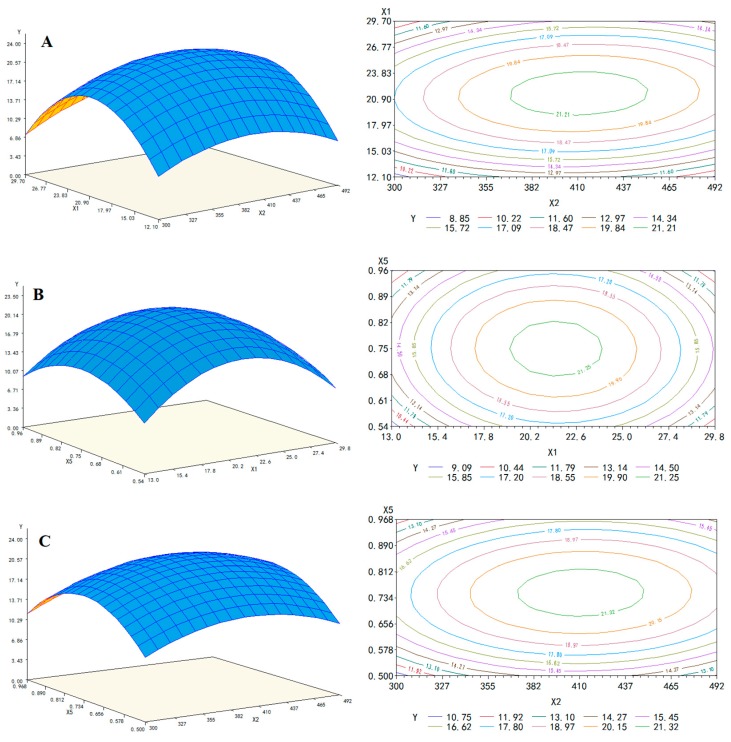
Response surface and contour map of total content of targets. (**A**) Ultrasonic power (X_2_) and liquid-solid ratio (X_1_) and, (**B**) liquid-solid ratio (X_1_) and methanol volume fraction (X_5_), and (**C**) ultrasonic power (X_2_) and methanol volume fraction (X_5_).

The response surface methodology is a mathematical statistical method for finding the optimal conditions in a multi-factor system. The Box–Behnken center combination design is the most common method to optimize multi-factors with three levels. The response surface graph is a surface map of the three-dimensional space formed via the response values for each factor (X_1_, X_2_, X_5_). The response surface analysis was performed according to the fitted regression equation, and the response surface and contour map of the interaction between variables was plotted Figure 1. A steeper slope for the response surface implies that the factor has a more significant impact on the response [34].

The contour line is oval, indicating that the interaction of the two factors is significant. The center point of the smallest ellipse in the contour is the highest or lowest point of the response surface. Figure 1A represents the influence of the liquid-solid ratio (X_1_) and ultrasonic power (X_2_) on the total yield as methanol concentration (X_5_) was held constant. These two variables affected the total yield of the 10 compounds, and the total yield first increased and then decreased with increasing liquid-solid ratio or ultrasonic power. However, the liquid-solid ratio curve was steeper, and the contour lines of the liquid-solid ratio were denser than those of ultrasonic power indicating that the liquid-solid ratio had a more significant effect on the total yield (Figure 1A).

Figure 1B showed that the impact of methanol concentration (X_5_) and liquid-solid ratio (X_1_) on the total yield and ultrasonic power (X_2_) remained relatively fixed. Similarly, it was not difficult to find that the total yield was affected by methanol concentration (X_5_) and liquid-solid ratio (X_1_). However, the total yield varied more for the liquid-solid ratio (X_1_) than that for methanol concentration (X_5_). Figure 1C showed the response surface and contour map representing interaction effects of methanol concentration (X_5_) and ultrasonic power (X_2_) on the total yield. The effect of methanol concentration (X_5_) on total yield was more significant than ultrasonic power (X_2_), as seen via the curve variation degree and the contour density. The optimal extraction conditions were predicted to be a liquid-solid ratio of 21.54:1 (mL/g), ultrasonic power of 410.46 W, and methanol solution of 74.97%, as determined by software SAS 9.2. The theoretical total yield of these 10 nutritional compounds was 21.90 μg/g under the optimum extraction condition.

Three verification experiments were performed under the optimal extraction conditions to verify the regression model. The optimum conditions were adjusted to liquid-solid ratio of 20:1 (mL/g), ultrasonic power of 410.0 W, and methanol solution of 75.0%, and considering the feasibility of actual operation. The average value (21.74 μg/g) of the verification experiments was almost the same as the predicted value—this proved that the model was effective and that the optimal extraction conditions were reliable. The optimal extraction conditions for these 10 nutrients were successfully obtained via the single factor design, Plackett–Burman design, and Box–Behnken design. The significance of each factor of Box–Behnken response surface design was the same as that of Plackett–Burman design in this study, which indicated that the results obtained could be mutually verified.

### 3.4. Method Validation

#### 3.4.1. Linearity, Limit of Detection, and Limit of Quantification

Mixed standard solutions with different concentrations of 10 nutritional compounds were examined by HPLC-MS/MS. Seven-point calibration curves were achieved with the use of optimal chromatographic peaks of the mixed standard solutions obtained under optimal instrumental condition [35]. The determination coefficients (*R*^2^) of the calibration equations ranged from 0.9990 to 0.9998 for these 10 target analytes (Table 2).

The limit of detection (LOD) is the lowest concentration of analyte that the instrument can detect, and limit of quantitation (LOQ) is the lowest concentration of analyte that can be quantitated. The LOD and LOQ were usually obtained by calculating the concentrations of target analytes at a signal-to-noise ratio (S/N) of 3-times and 10-times, respectively. Table 2 shows that the LODs and LOQs for 10 nutritional compounds by this method were 0.01–0.11 µg/kg and 0.04–0.34 µg/kg, respectively. The low LODs and LOQs for the 10 nutritional compounds in black and white sesame seeds confirmed the sensitivity of the method.

#### 3.4.2. Matrix Effect (ME)

In chemical analysis, the matrix refers to the components other than the target analyte in the sample. The matrix effects were primarily due to analyte ionization efficiency. The matrix could reduce or increase the ion intensity of the analytes and might significantly influence the reproducibility and accuracy of the analysis. Matrix effects were often examined by adopting the standard curve method and the standard additions method [36].

The key ways to reduce the matrix effect included improving sample pretreatment and purification, the matrix standard solution calibration method, and considering that the chemical properties of the analytes could affect the ion source response [37]. Due to the lack of blank matrices for sesame seeds, matrix effects were examined by using a quality control sesame sample with known concentrations for the 10 nutritional compounds. Three sets of experiments were designed to evaluate the matrix effects of the method including the standard additions (A (matrix + standard)), matrix alone (B (matrix)), and standard only (C (standard)). MEs were calculated for 10 nutritional compounds in sesame seeds using the following equation [38]:

ME (%) = [(A − B)/C − 1]*100%.(4)

The MEs data are shown in Table 3. The MEs of all target compounds ranged from −13.8% to 11.2%, which indicated that the method had no significant matrix effects for all cases.

#### 3.4.3. Precision

Precision was assessed by repeated measurements. Intra-day precision and inter-day precision were investigated via the percent relative standard deviation (RSD%) of quality control sample spiked with three concentrations in one day (*n* = 3) and five consecutive days (*n* = 5) under the same conditions. The intra-day precisions and inter-day precisions for 10 nutritional compounds were lower than 9.6% and 12.9%, respectively (Table 3). These results indicated that these 10 antioxidants have good stability.

#### 3.4.4. Recovery

A recovery experiment was performed by adding the standard solutions at three different concentrations (50 µg/kg, 200 µg/kg, and 500 µg/kg) in sesame seed samples. The recoveries were calculated by adopting the ratio of amount subtracting the sample original amount from the total measured amount over the spiked amount to spiked amount, and expressed as the percent recovery. Table 3 summarized the acceptable recovery values seen here (71.1% to 118.3%) for all target compounds. The recoveries of these 10 compounds in sesame seeds samples indicated that the method was accurate, stable, and reliable.

Accurate measurement of these nutrients is critical for the assessment of sesame and its byproducts. The analytical method for detecting vitamin B_2_ is mainly LC with capillary optical fiber laser-induced fluorescence. Kiba et al. determined N-acetyl-l-leucine via enzyme liquid reactor-based high-performance liquid chromatography [39]. The reagents could be recycled, and the reagent consumption was low, but the poor stability resulted in a frequent renewal of the enzyme reactor. There are few reports on coniferyl aldehyde. Coniferyl aldehyde was separated in soybean roots at 12.52 min via HPLC-CAD at 340 nm [40]. The method is simple and fast, but with low sensitivity. Wang et al. conducted pharmacokinetic studies of phloretin using LC-MS/MS, which had a low limit of quantitation of 20 ng/mL, but the pretreatment was slightly more complicated [41]. Densitometric thin layer chromatographic analysis was used to determine the l-hyoscyamine in sputum. This method was reliable but required derivatization via the Dragendorff reagent [42]. 8-methoxypsoralen and 5-methoxypsoralen were simultaneously determined in cigarettes via gas chromatography-mass spectrometry with solid-phase extraction [25]. However, the cost of the process is markedly increased via the use of the solid phase extraction column for pretreatment. Since these 10 compounds described above are quite diverse, the simultaneous analysis has not yet been reported. The simultaneous quantitation of these 10 antioxidants was achieved for the first time by using the proposed UAE-HPLC-MS/MS method.

### 3.5. Application to Sesame Seeds Samples

The validated UAE-HPLC-MS/MS method was next used for the simultaneous determination of these 10 nutritional compounds in sesame seeds. Figure S2 showed that the 10 compounds could be quickly separated and detected within 7 min. The statistical analysis was performed on all target compounds in sesame seeds via the MetaboAnalyst 4.0 platform. A volcano plot was obtained by taking the negative logarithm of the *p*-value (-Log10 (*p*-value)) and the logarithm of the fold change (log2 (fold change)) as the vertical and horizontal coordinates, respectively.

**Figure 2:**
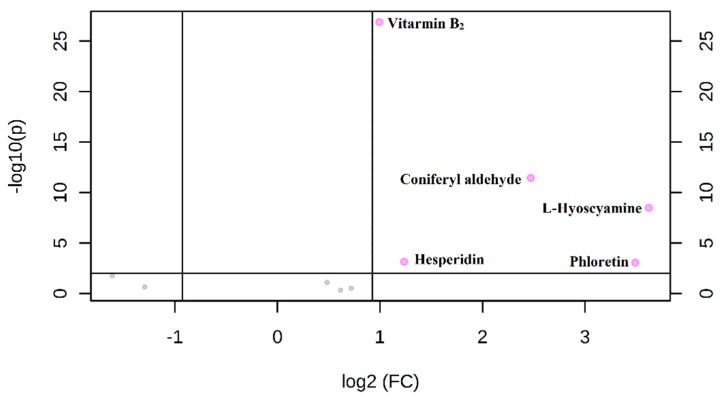
Volcano plot of ten targets in white and black sesames.

Concentration differences for the nutritional compounds between black and white sesame seeds were identified using the *p*-value and fold change (Figure 2). The red labeled dots represented compounds that are significantly different (*p* < 0.01, fold change > 1.9) in Figure 2. The results showed that the content of vitamin B_2_, phloretin, hesperidin, l-hyoscyamine, and coniferyl aldehyde was significantly different between black and white sesame seeds. The specific differences were presented in the box-plot in Figure S3. The contents of five compounds were significantly higher in black sesame seeds than in white sesame seeds. The contents of five nutrients are further detailed in Table 4 (other compounds data were not shown). The content of coniferyl aldehyde, l-hyoscyamine, and phloretin in black sesame seeds was much higher than that of white sesame seeds—these can be used as biomarkers of black sesame seeds. The nutrient differences between black and white sesame seeds can provide a theoretical basis for the evaluation of sesame quality and the cultivation of high-quality sesame varieties.

## 4. Conclusions

This work used the single factor experiment and Plackett–Burman test to identify the three factors that significantly affect the ultrasonic-assisted extraction of target compounds: methanol concentration, ultrasonic power, and liquid-to-solid ratio. This was done with a first-order kinetic equation. A quadratic multiple regression model of the three independent variables was then established using Box–Behnken design. The significant factors were optimized by analysis of variance and response surface analysis leading to a significant and reliable regression equation model.

The optimal conditions for the extraction of target nutrients in sesame seeds include methanol concentration of 75.0%, liquid-to-material ratio of 20:1 (mL/g), extraction temperature of 50 °C, extraction power of 410.0 W, extraction time of 65 min, and one round of extraction; the total yield of targets is 21.74 μg/g under these conditions. The mobile phase used was 0.1% formic acid in acetonitrile and 0.1% formic acid in water, and the column was a Thermo Syncronis C18 reverse phase column (100 mm × 2.1 mm, 3 μm). All target analytes only need one injection and could be separated and measured quickly (<7 min). Simultaneous extraction and detection of 10 different types of nutritional compounds participated in multiple metabolic pathways were achieved. This method had favorable linearity, good recoveries, intra-day and inter-day precisions, negligible matrix effects, and low LODs and LOQs for these 10 nutritional compounds. This study is of great value for the selection and improvement of high nutritional quality sesame varieties, the development of functional products, and the promotion of human health.

## Figures and Tables

**Table 1:** Mass spectrometric parameters of target analytes.

Name	Retention Time/min	Parent Ion	Product Ion	Collision Energy/eV	Ion Polarity	Tube Lens/V
d-Homoproline	0.83	130.132	84.370 > 56.427	14/32	+	86
Vitarmin B_2_	3.81	377.066	243.233 > 172.283	22/36	+	123
l-Hyoscyamine	3.88	290.100	124.303 > 93.400	22/31	+	98
N-Acetyl-l-leucine	4.21	174.110	86.398 > 128.294	16/7	+	86
Hesperidin	4.28	611.160	303.232 > 177.324	21/34	+	142
Ferulic acid	4.48	193.020	134.060	16	−	62
Coniferyl aldehyde	4.86	179.065	147.226 > 91.311	26/13	+	88
Phloretin	5.26	275.026	107.282 > 77.372	20/46	+	103
5-Methoxypsoralen	5.80	217.100	202.156 > 89.343	21/46	+	94
8-Methoxypsoralen	6.06	217.100	202.156 > 174.183	20/29	+	92

**Table 2:** Linear equation, determination coefficients (*R*^2^), LODs and LOQs of target compounds.

Analytes	Linear Range (μg/kg)	Linear Equation	*R*^2^ Value	LOD (μg/kg)	LOQ (μg/kg)
d-Homoproline	0.1–2000	y = 43990x − 711749	0.9995	0.05	0.16
Vitarmin B_2_	0.1–1800	y =15838x − 408323	0.9993	0.03	0.09
l-Hyoscyamine	0.1–1500	y = 91957.5x + 12035	0.9996	0.01	0.04
N-Acetyl-l-leucine	1.0–1800	y = 4045.59x − 173347	0.9993	0.11	0.34
Hesperidin	1.0–2000	y = 3213.93x − 44406.7	0.9998	0.02	0.07
Ferulic acid	50.0–2000	y = 15.2178x − 3105.91	0.9998	0.02	0.06
Coniferyl aldehyde	10.0–1800	y = 5845.91x − 143573	0.9993	0.08	0.25
Phloretin	0.5–1500	y = 4079.1x − 25758	0.9996	0.07	0.21
5-Methoxypsoralen	0.5–1800	y = 6620.08x − 61279	0.9997	0.08	0.24
8-Methoxypsoralen	0.5–2000	y = 35504.8x − 951479	0.9990	0.08	0.25

**Table 3:** Intra-day precisions, inter-day precisions, matrix effects and recoveries of 10 nutritional compounds.

Analytes	Intra-Day Precision (RSD %, *n* = 3)	Inter-Day Precision (RSD %, *n* = 5)	Recovery (%, *n* = 3)	Matrix Effect (%, *n* = 3)
-	50/200/500 μg/kg	50/200/500 μg/kg	50/200/500 μg/kg	50/200/500 μg/kg
d-Homoproline	3.6/1.9/4.6	6.7/3.9/4.1	117.7/114.5/118.3	8.2/9.4/11.2
Vitamin B_2_	2.2/7.7/3.6	8.1/9.2/10.4	71.1/78.6/73.5	−13.8/−9.2/−10.3
l-Hyoscyamine	1.9/3.2/4.4	5.6/3.3/8.2	80.9/89.1/104.8	−6.4/−5.1/−3.8
N-Acetyl-l-leucine	5.8/8.7/9.6	6.3/7.1/8.4	100.8/109.1/90.2	−4.5/−3.9/−8.1
Hesperidin	5.4/4.9/3.7	5.5/4.9/9.4	82.4/79.3/89.5	−10.8/−7.4/−6.9
Ferulic acid	4.3/2.8/5.9	7.6/5.9/8.7	95.9/103.4/109.1	9.1/6.3/8.5
Coniferyl aldehyde	3.8/5.7/7.9	8.6/4.2/9.6	99.6/106.3/110.4	6.8/5.8/9.4
Phloretin	6.7/7.8/8.4	6.5/9.4/12.9	97.7/98.6/105.3	−5.4/−4.6/−5.2
5-Methoxypsoralen	3.9/5.8/7.4	8.8/5.9/6.3	107.5/110.8/115.1	8.6/9.0/10.6
8-Methoxypsoralen	4.8/3.3/8.0	7.1/6.4/4.9	89.6/96.2/107.4	5.5/4.9/8.7

**Table 4:** Content of 5 differential nutrients in sesame seeds.

Analytes	Black Sesame Seeds/(mg/kg)	White Sesame Seeds/(mg/kg)
Vitamin B_2_	0.10 ± 0.01	0.05 ± 0.01
l-Hyoscyamine	0.53 ± 0.08	0.04 ± 0.01
Hesperidin	0.13 ± 0.01	0.06 ± 0.01
Coniferyl aldehyde	1.99 ± 0.56	0.41 ± 0.15
Phloretin	5.05 ± 0.75	0.47 ± 0.09

## Supplementary Materials

The following are available online at https://www.mdpi.com/2076-3921/8/8/321/s1, Figure S1: Results of preliminary screening on six general parameters, Figure S2: LC-MS/MS chromatograms obtained for the standard solution of 10 targets, Figure S3: Box plot of differential compounds, Table S1: Factors and coded value of Plackett-Burman design, Table S2: Design matrix and results of Plackett-Burman test, Table S3: Factors and coded value of Box-Behnken design, Table S4: Design matrix and results of Box-Behnken test, Table S5: Analysis results of Plackett-Burman design, Table S6: Analysis results of Box-Behnken design.
