# Supplementary material for: Optimization of an Ultrasound-Assisted Extraction for Simultaneous Determination of Antioxidants in Sesame with Response Surface Methodology
Source: Antioxidants (Basel). 2019 Aug 19;8(8):321. doi: 10.3390/antiox8080321 (PMC6720519; doi:10.3390/antiox8080321)
Supplement: Supplementary file 1 [file antioxidants-08-00321-s001.pdf]

Optimization of an Ultrasound-assisted extraction for simultaneous determination of antioxidants in sesame with response surface methodology

Dandan Wang[1,2,†], Liangxiao Zhang[1,3,4,†], Yueqing Xu[1,2], Xin Qi[1,4], Xuefang Wang[1,4], Xiupin Wang[1,4], Qi Zhang[1,5], Peiwu Li[1,3,4,5,*]

[1] Oil Crops Research Institute, Chinese Academy of Agricultural Sciences, Wuhan 430062, China

[2] Key Laboratory of Biology and Genetic Improvement of Oil Crops, Ministry of Agriculture and Rural Affairs, Wuhan 430062, China

[3] Laboratory of Quality and Safety Risk Assessment for Oilseed Products (Wuhan), Ministry of Agriculture and Rural Affairs, Wuhan 430062, China

[4] Quality Inspection and Test Center for Oilseed Products, Ministry of Agriculture and Rural Affairs, Wuhan 430062, China

[5] Key Laboratory of Detection for Mycotoxins, Ministry of Agriculture and Rural Affairs, Wuhan 430062, China

[†] These authors contributed equally to this study

*Corresponding authors: prof. Peiwu Li at Oil Crops Research Institute, Chinese Academy of Agricultural Sciences, Wuhan 430062, China

Tel.: +86 27 86812943; Fax: +86 27 86812862

E-mail addresses: Peiwuli@oilcrops.cn (P. Li)

Supplementary Materials

Figure S1 Results of preliminary screening on six general parameters

Figure S2 LC-MS/MS chromatograms obtained for the standard solution of 10 targets

Figure S3 Box plot of differential compounds

Table S1 Factors and coded value of Plackett-Burman design

Table S2 Design matrix and results of Plackett-Burman test

Table S3 Factors and coded value of Box-Behnken design

Table S4 Design matrix and results of Box-Behnken test

Table S5 Analysis results of Plackett-Burman design

Table S6 Analysis results of Box-Behnken design

Figure S1 Results of preliminary screening on six general parameters

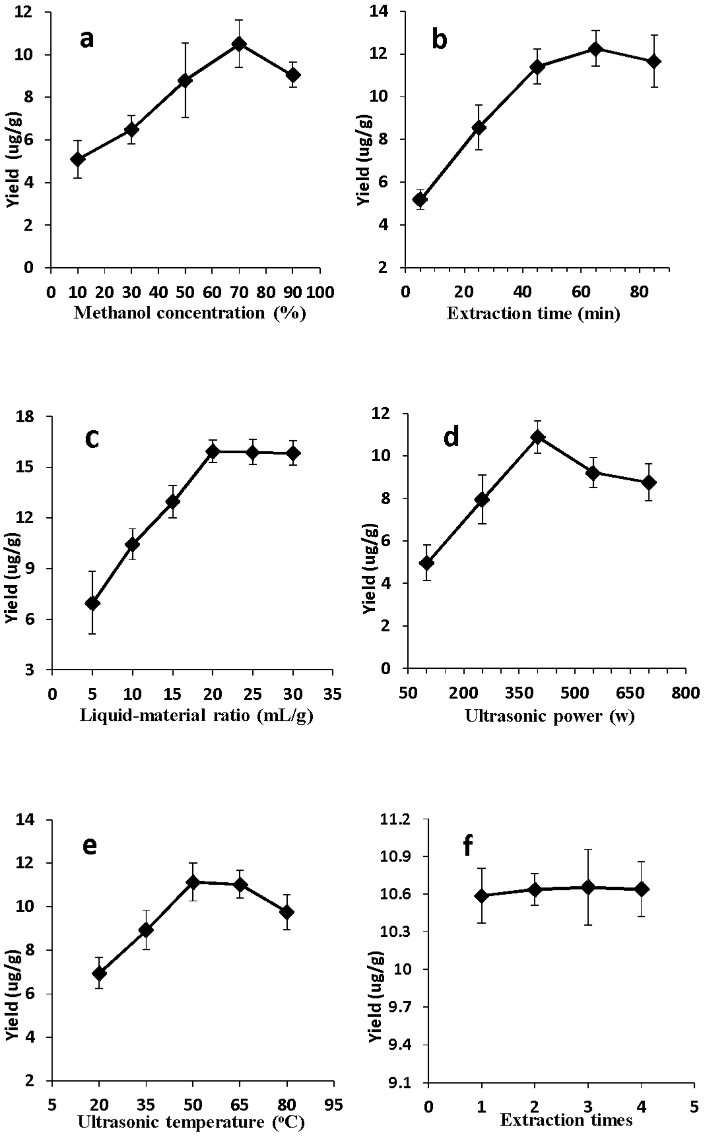

Figure S2 LC-MS/MS chromatograms obtained for the standard solution of 10 targets

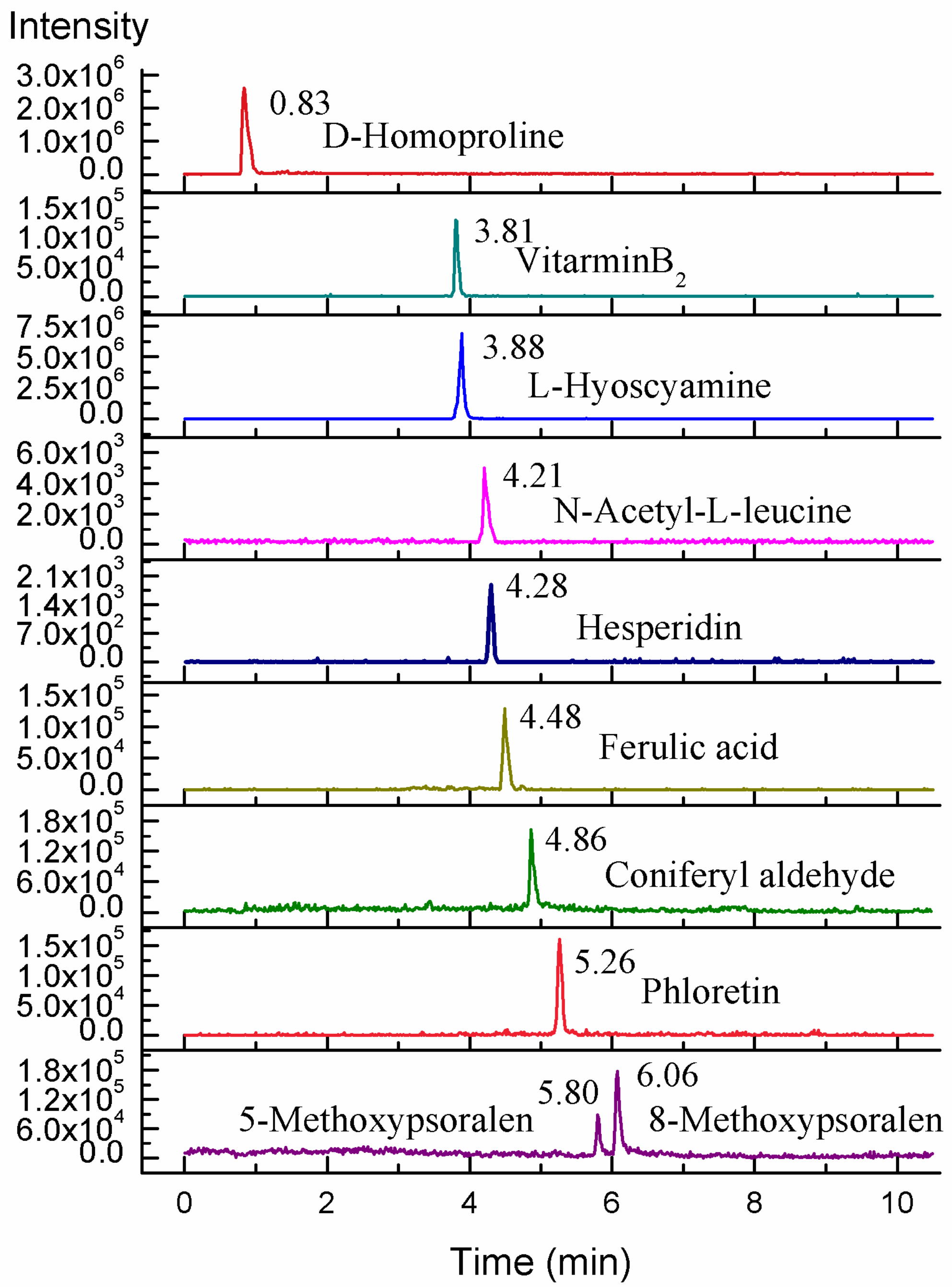

Figure S3 Box plot of differential compounds

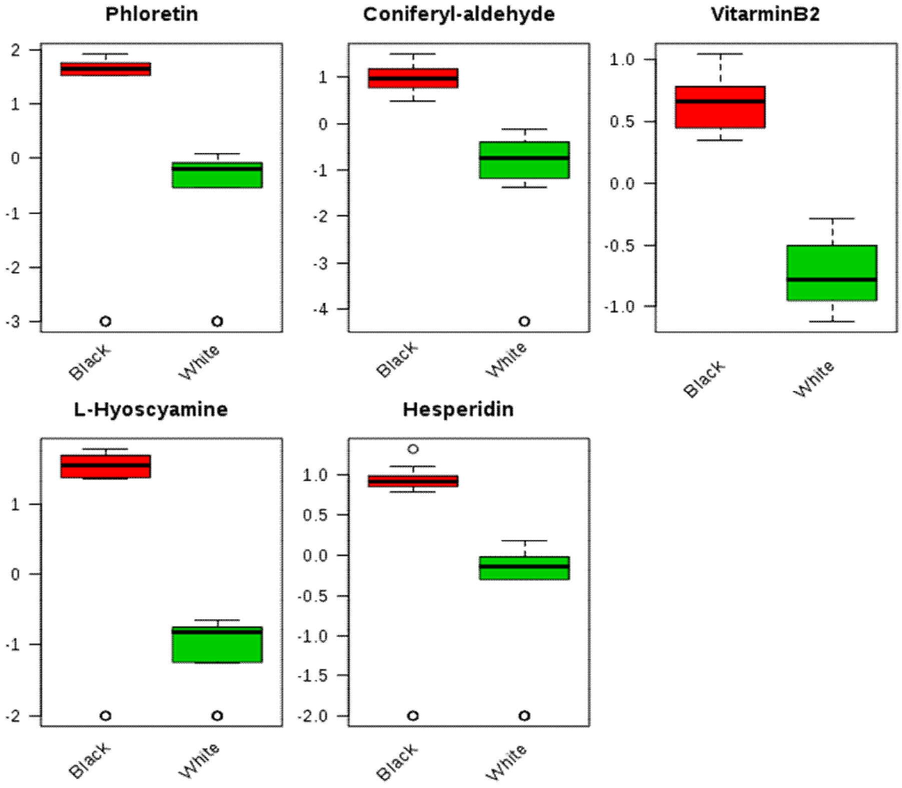

Table S1 Factors and coded value of Plackett-Burman design

Term	Variable	Coded level	
		-1	1
X_1	liquid-solid ratio (mL/g)	15/1	25/1
X_2	ultrasonic power (w)	300	500
X_3	ultrasonic time (min)	35	55
X_4	ultrasonic temperature (°C)	40	60
X_5	methanol volume fraction (%)	70	90
X_6	extraction times	1	3

Table S2 Design matrix and results of Plackett-Burman test

Run	Liquid-solid ratio (X_1)	Ultrasonic power (X_2)	Ultrasonic time (X_3)	Ultrasonic temperature (X_4)	Methanol volume fraction (X_5)	Extraction times (X_6)	Total actual yield （ug/g）
1	-1	-1	-1	-1	1	1	9.265
2	1	1	-1	1	1	1	9.530
3	1	-1	-1	1	-1	-1	17.518
4	-1	-1	1	1	1	1	10.183
5	1	1	1	-1	1	-1	9.346
6	-1	1	-1	1	1	-1	9.095
7	-1	1	-1	-1	-1	-1	11.596
8	-1	1	1	-1	-1	1	11.959
9	1	-1	-1	-1	-1	1	17.888
10	1	1	1	1	-1	1	16.433
11	1	-1	1	-1	1	-1	11.165
12	-1	-1	1	1	-1	-1	13.915

Table S3 Factors and coded value of Box-Behnken design

Term	Variable	Coded level		
		-1	0	1
X_1	liquid-solid ratio (mL/g)	15/1	20/1	25/1
X_2	ultrasonic power (w)	300	400	500
X_5	methanol volume fraction (%)	70	90	90

Table S4 Design matrix and results of Box-Behnken test

Run	Liquid-solid ratio (X_1)	Ultrasonic power (X_2)	Methanol volume fraction (X_5)	Total actual yield (ug/g)
1	-1	-1	0	12.322
2	1	-1	0	15.381
3	-1	1	0	12.896
4	1	1	0	17.828
5	-1	0	-1	17.303
6	1	0	-1	19.937
7	-1	0	1	15.013
8	1	0	1	17.326
9	0	-1	-1	16.694
10	0	1	-1	17.912
11	0	-1	1	14.298
12	0	1	1	15.977
13	0	0	0	21.135
14	0	0	0	21.537
15	0	0	0	21.308

Table S5 Analysis results of Plackett-Burman design

Regression data					
**Variable**	**Degree of freedom**	**Parameter Estimate**	**Standard Error**	**t Value**	**Pr > |t|**
Intercept	1	12.32442	0.30139	40.89	0.0005**
X_1	1	1.32225	0.30139	4.39	0.0071**
X_2	1	-0.99792	0.30139	-3.31	0.0212*
X_3	1	-0.15758	0.30139	-0.52	0.6234
X_4	1	0.45458	0.30139	1.51	0.1918
X_5	1	-2.56042	0.30139	-8.50	0.0004**
X_6	1	0.21858	0.30139	0.73	0.5008
**Analysis of Variance**					
**Source**	**Degree of freedom**	**Sum of squares**	**Mean square**	**F Value**	**Pr > F**
Model	6	114.95008	19.15835	17.58	0.0032**
Error	5	5.45002	1.09000		
Corrected Total	11	120.40010			
Root MSE	1.04403	R-Square	0.9547		
Dependent Mean	12.32442	Adj R-Sq	0.9004		
Coeff Var	8.47126				

*,** represent significant differences (P<0.05) and extremely significant differences (P<0.01), respectively.

Table S6 Analysis results of Plackett-Burman design

Source	Degree of freedom	Sum of squares	Mean Square	F Value	Pr > F
Linear	3	35.95549	0.2994	46.74	0.0004**
Quadratic	3	81.893687	0.6820	106.46	<.0001**
Crossproduct	3	0.955923	0.0080	1.24	0.3870
Total Model	9	118.805159	0.9893	51.48	0.0002**
Lack of Fit	3	1.200776	0.400259	9.84	0.0936
Pure Error	2	0.081325	0.040662		
Total Error	5	1.282101	0.256420		
**Root MSE**	0.506380	**R-Square**	0.9893	**Coefficient of Variation**	2.9571
**Variable**	**Degree of freedom**	**Parameter Estimate**	**Standard Error**	**t Value**	**Pr > |t|**
Intercept	1	-151.965333	22.074508	-6.88	0.0010**
X_1	1	4.513583	0.619841	7.23	0.0008**
X_2	1	0.295224	0.030992	9.53	0.0002**
X_5	1	172.533333	44.566674	3.87	0.0117*
X_1*X_1	1	-0.110908	0.010541	-10.52	<.0001**
X_2*X_1	1	0.000936	0.000506	1.85	0.1236
X_2*X_2	1	-0.000395	0.000026353	-14.98	<.0001**
X_5*X_1	1	-0.160500	0.506380	-0.32	0.7641
X_5*X_2	1	0.011525	0.025319	0.46	0.6681
X_5*X_5	1	-115.920833	26.352827	-4.40	0.0070**

*,** represent significant differences (P<0.05), extremely significant differences (P<0.01), respectively; F listed value (95%), $F_{9,5}$ = 3.63.
